# Impact of Heated Tobacco Products, E-Cigarettes, and Combustible Cigarettes on Small Airways and Arterial Stiffness

**DOI:** 10.3390/toxics11090758

**Published:** 2023-09-06

**Authors:** Isabel Goebel, Theresa Mohr, Paul N. Axt, Henrik Watz, Frederik Trinkmann, Markus Weckmann, Daniel Drömann, Klaas F. Franzen

**Affiliations:** 1 Medical Clinic III, Site Lübeck, University Hospital Schleswig-Holstein, 23538 Lübeck, Germany; 2 Airway Research Center North (ARCN), DZL, 35392 Lübeck, Germany; 3 Pulmonary Research Institute (PRI) at LungenClinic Großhansdorf, 22927 Großhansdorf, Germany; 4 Thoraxklinik Heidelberg, Translational Lung Research Center Heidelberg (TLRC), German Center for Lung Research (DZL), University Hospital Heidelberg, 69126 Heidelberg, Germany; 5 Department of Biomedical Informatics, Center for Preventive Medicine and Digital Health (CPD), University Medical Center Mannheim, Heidelberg University, 68167 Mannheim, Germany; 6 Clinic for Pediatric, Site Lübeck, University Hospital Schleswig-Holstein, 23538 Lübeck, Germany

**Keywords:** heated tobacco products, IQOS, GLO, e-cigarette, occasional smoking, small airway function, arterial stiffness, central blood pressure, smoking cessation, nicotine replacement therapy

## Abstract

Smoking cessation is difficult but maintaining smoke-free without nicotine replacement therapy is even harder. During the last few years, several different alternative products, including heated tobacco products (HTP), have been introduced to the market. In this study, we investigated the acute effects of IQOS^TM^ and glo^TM^ (two HTP) consumption on small airway function and arterial stiffness in a head-to-head design, comparing them to combustible cigarettes, nicotine-free e-cigarettes and a sham smoking group. Seventeen healthy occasional smokers were included in a single-center, five-arm, crossover study. The parameters of small airway function and hemodynamics were collected at several time points before and after consumption using Mobil-O-Graph™ (I.E.M., Stolberg, Germany) and TremoFlo^®^ c-100 (THORASYS Thoracic Medical Systems Inc., Montreal, QC, Canada). Small airway obstruction and resistance were both significantly increased after the consumption of cigarettes and substitute products. All products containing nicotine led to similar significant increases in blood pressure and arterial stiffness. Hemodynamic parameters were also increased after the consumption of e-cigarettes without nicotine, but compared to nicotine-containing products, the increase was shorter and weaker. We conclude that, although it has yet to be determined why, HTP have acute harmful effects on small airway function, possibly even exceeding the effects of combustible cigarettes. Like other nicotine-containing products, HTP leads to a nicotine-related acute increase in arterial stiffness and cardiovascular stress, similar to combustible cigarettes, which associates these products with an increased cardiovascular risk.

## 1. Introduction

Smoking is the most important avoidable health risk in Germany. Every year more than 127,000 deaths can be attributed to smoking-related diseases [1]. While until 2020, the percentage of active smokers in Germany decreased steadily, since 2021, the numbers have increased again, with them at 35.5% in December 2022 [2].

In total, 39% of German active smokers would like to stop smoking [3]. Unfortunately, long-term smoking cessation without substitution is effective only in 3–5% [4]. With nicotine-replacement therapy (NRT, products giving small steady doses of nicotine to stop nicotine cravings, easing the transition to complete nicotine abstinence), quitting rates can be raised by 50–70% [5]. Recently, e-cigarettes, which do not count as NRT, were shown to reduce recidivism rates better than smoking cessation without substitution [6,7]. However, various studies suggest that the consumption of e-cigarettes is a health risk itself [8,9,10], emphasizing the need for alternative products that are both satisfying and cause as little harm as possible. In addition, e-cigarettes are intensively discussed for smoking cessation, because a lot of medical societies do not recommend them for smoking cessation. In recent years, several cigarette alternatives, among them heated tobacco products (HTP), have been introduced to the market. In 2017 sales of IQOS^TM^ from Philipp Morris International (PMI) were launched in Germany, and GLO^TM^ from British American Tobacco followed in 2020. These relatively new devices promise to be less harmful to both consumers and bystanders [11,12]. Instead of burning tobacco to inhale the nicotine, the electric heating systems aerosolize nicotine from tobacco without any combustion taking place. The combustion in conventional cigarettes happens at around 600 °C and above, producing smoke that contains nicotine but also an abundance of hazardous and carcinogenic substances [13,14]

In HTP, temperatures vary between different products but generally are below 350 °C [15]. In IQOS^TM^ tobacco is heated at an average of 201 °C, the GLO^TM^ uses even lower temperatures around 170 °C [16]. Hence, these aerosols supposedly contain fewer unwanted substances [17]. Nevertheless, sufficient evidence that HTP are less harmful than conventional cigarettes is still missing [18]. Furthermore, studies indicate that HTP might not be as low risk as promoted by manufacturers [16,17,19]. In animal models, evidence could be found that HTP lead to an inflammatory response in the lung, causing lung emphysema, which is the remodeling of small and large airways, and activating pathways that are also involved in tobacco smoke-induced carcinogenesis [20,21,22]. Arterial stiffness parameters were also shown to be increased after HTP consumption [23,24], underlining that further research on the impact of these devices is needed.

Therefore, in this study, we investigated the acute effects on arterial stiffness and small airway function in two heated tobacco products (IQOS^TM^ and GLO^TM^) and compared them to combustible cigarettes, e-cigarettes without nicotine, and sham smoking in a head-to-head design.

## 2. Materials and Methods

### 2.1. Study Cohort and Design

In this single-center, five-arm cross-over study, 17 healthy occasional smokers were included. The participants were recruited from students at the University of Lübeck. Before inclusion, the participants were screened for exclusion criteria: (i) non-smokers; (ii) obesity; (iii) pregnancy; (iv) mental disorders; (v) cardiovascular disease; (vi) pulmonary disease; (vii) age < 18 years; and (viii) abnormal physical examination. In alliance with the guidelines for the measurement of arterial stiffness [25], the participants were instructed to neither drink alcohol nor to consume any products containing nicotine 48 h prior to every measurement. Test days were scheduled at least 48 h apart to provide a washout period. After at least 24 h of consideration time, a consent form was signed by both participant and examiner. The local ethics committee included the study on DRKS (DRKS00020446).

**Figure 1:**
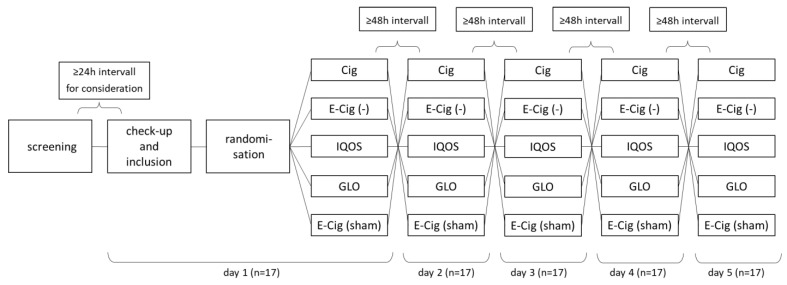
Flowchart and study design for each test person. Study design for each test person. Randomization is performed on day 1, determining the order of the test devices. Every device is tested once.

The five study arms are composed of (a) combustible cigarette (Cig) (Marlboro Gold 0.5 mg/cigarette), (b) e-cigarette without nicotine (E-Cig (-)) (DIPSE-eGo-cigarette; 0 mg/mL, tobacco flavor), (c) heated tobacco product “IQOS”, (d) heated tobacco product “GLO”, and (e) sham smoking, vaping with an e-cigarette without liquid (DIPSE-eGo-cigarette; 0 mg/mL). Nicotine-free e-cigarettes have been used in previous studies as a control group [24]. Since they still contain various inhalable substances that could affect lung function and arterial stiffness, we also included the sham smoking group, in which an empty e-cigarette was used to mimic smoking, but nothing is inhaled. Figure 1 displays the flowchart of the study.

The order of the test devices was drawn by lot. Every device was only used once by each test person, and in order to be taken into analysis, all four devices must have been assessed (CIG, E-cig (-), IQOS, GLO). Sham smoking was tested in 7 out of the 17 participants and was also included in randomization. Measurements were performed at the same time of the day, to rule out systematic errors caused by circadian rhythm. To grant standardized conditions, test persons were instructed in a smoking scheme for every device. Heated tobacco products (IQOS^TM^ and GLO^TM^) were to be consumed as recommended in the instruction manual [26,27]. Cigarettes were to be completely smoked. E-cigarettes without nicotine and empty e-cigarettes for sham smoking were to be vaped ten times for 3–5 s with a rest period of 30 s in between [28].

Baseline cardiovascular measurements were performed with Mobil-O-Graph™ (I.E.M., Stolberg, Germany) in 5 min intervals starting 30 min prior to the intervention until 60 min post intervention. TremoFlo^®^ c-100 (THORASYS Thoracic Medical Systems Inc.) was used for pulmonary measurements two times prior to the intervention and 5, 10, 15, 30, and 60 min afterwards.

### 2.2. Measurement of Resistance and Reactance in Central and Small Airways

Lung function analysis was performed using TremoFlo^®^ c-100 (THORASYS Thoracic Medical Systems Inc., Montreal, QC, Canada). Hereby, airwave oscillometry (AO) with oscillation frequencies between 5 and 37 Hz was used. Since this frequency is higher than the tidal breathing frequency, a high-frequency wave was superimposed onto the breathing waveform, allowing the device to calculate resistance (R) and reactance (X) [29].

To allow the oscillatory waves to pass directly through the trachea, the test person needed to be in a seated, upright position, with the head slightly extended. They also need a nose clip to seal the nasal passage, with their hands supporting their cheeks on both sides and their lips enclosing the mouthpiece. Three consecutive 20 s. measurements during tidal breathing were performed. The average was used for analysis. The value obtained prior to the intervention, referred to as the baseline value, was used as a reference. For quality control reasons, the coefficient of variation (CV) ought to be lower than 15% [29].

Resistance is defined as the energy needed to move the pressure wave through the airways [30]. It is determined at 5 and 19 Hz, representing the total (R5) and central (R19) airway obstruction. R5–R19 is the calculated difference of the total airway resistance (R5) and central airway resistance (R19), representing the peripheral airway obstruction. Reactance is a complex term representing the energy determined by flow airflow dynamics in the airways, influenced by the elasticity of tissue and the interstitial forces [30]. AX is defined as the reactance area. The increased values are correlated with distal obstruction [30]. The tidal volume (VT) represents the ventilated air volume during physiological in- and expiration [31].

### 2.3. Measurement of Peripheral and Central Blood Pressure and Arterial Stiffness

In this study, Mobil-O-Graph^TM^ (software version HMS CS 4.2, I.E.M. GmbH, Stolberg, Germany) was used to measure hemodynamic parameters. This device performs pulse wave analysis, using an oscillometric measuring technique. With a common cuff peripheral blood pressure and heart rate were detected. Subsequently, waveforms of the brachial artery were recorded at diastolic blood pressure level. The ARCSolver algorithm then estimated the central systolic blood pressure and central waveforms, allowing pulse waveform analysis and the calculation of arterial stiffness parameters to occur, such as pulse pressure, augmentation index, and total peripheral resistance [32,33].

Measurements were performed while the test persons subjects were seated. A standard blood pressure cuff was placed on the upper arm at the right atrial level. The measurement prior to the intervention, referred to as baseline value, was used as a reference.

### 2.4. Statistical Analysis

SPSS statistical software (SPSS 23 Inc., Chicago, IL, USA) was used for statistical analyses and Graph Pad Prism (Graph Pad Prism 6.01 for Windows, Graph Pad Software, San Diego, CA, USA) was used for graph editing. For the statistical references of blood pressure, heart rate, arterial stiffness parameters, lung resistance, and reactance, the baseline mean values were applied. The measurement prior to the intervention was referred to as the baseline value. All the above-mentioned processes were tested for normal distribution by Kolmogorov–Smirnov tests. The crossover design was used as the basis for the decision to calculate a two-way repeated measures ANOVA to estimate an interaction between the type of device used and time. If an interaction was found, a post hoc test (Bonferroni) was applied. Student’s *t*-test was used to evaluate differences between continuous baseline characteristics between groups. To individually analyze differences at various time points between the four devices, ANOVA was used. Where appropriate, a multivariate analysis of variance (MANOVA) was applied to level age, mean arterial pressure (MAP), heart rate (HR), and sex. All data are expressed as mean ± standard deviation (SD) unless otherwise specified. An alpha error below 5% was considered statistically significant.

## 3. Results

All parameters were taken prior to the intervention as well as up to 60 min after the intervention while test persons were resting. Hemodynamic parameters were hereby taken in 5 min intervals, starting 10 min before the intervention. Lung function parameters were taken 20 and 10 min before the intervention and 5, 15, 30, and 60 min after the intervention. Peak nicotine levels of inhalative nicotine consumption can be found after 5–10 min and decline rapidly for 20 min after consumption due to tissue distribution [34]. Therefore, we paid special attention to the first 20 min when analyzing hemodynamic parameters. For clarity, the following abbreviations were used when describing the groups: IQOS^TM—^IQOS, glo^TM^—GLO; cigarette—Cig; e-cigarette without nicotine—E-Cig (-); and sham smoking—Sham. The measurement prior to the intervention (−1 min in hemodynamic parameters and −10 min in respiratory parameters) is used as a baseline value that the post-intervention measurements are compared to. In the following, *p* < 0.05 (*) is considered significant, *p* < 0.01 (#) is considered highly significant.

### 3.1. Baseline Characteristics

In this study, we included 17 healthy participants with a mean age of 24.2 ± 1.1 years. Test persons were healthy and normal weight. Test persons started to smoke occasionally. On average they smoked 1.12 ± 0.8 cigarettes per day; however, all participants scored 0 on the Fagerström Test for nicotine dependence. Gender-specific baseline characteristics are shown in Table 1. All 17 test subjects tested all devices (Cig, E-cig (-), IQOS, GLO), and Sham was tested in 7 test subjects.

### 3.2. Respiratory Parameters

**Figure 2:**
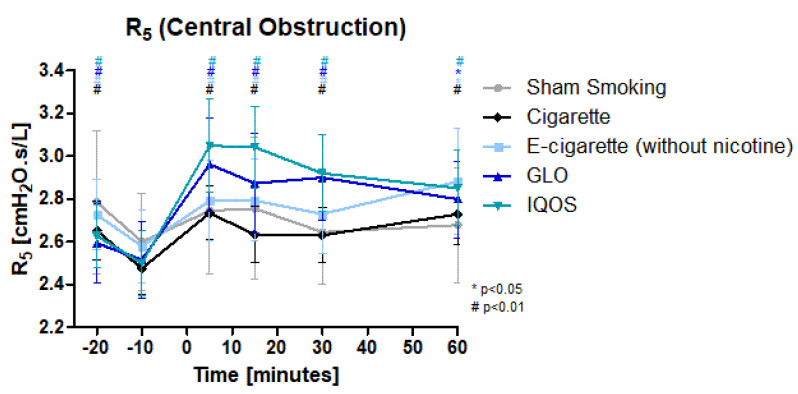
R5 (Central obstruction): R5 as a marker of central obstruction is displayed over time. Values are normally distributed. *t*-tests were performed, comparing each value to the baseline value (−10 min) of the respective group.

Increased central and peripheral obstruction as well as reactance was detected in all groups but sham smoking. Central obstruction (Figure 2) was significantly increased 5 min after the intervention in all devices. (Cig, E-Cig (-), IQOS, and GLO). The maximum increase was reached after 5 min in Cig, IQOS, and GLO, after 15 min in the sham smoking group and after 60 min in the E-cig (-) group. The initial and total increase of IQOS (22%) and GLO (17.7%) was higher than in Cig (10.5%), E-Cig (-) (8.3%/11.7%), and sham smoking (5.6%/6%). Significant elevation lasted until 60 min post intervention (Cig and IQOS highly significant, E-Cig (-) and GLO significant). No significant changes were detected in the sham smoking group.

**Figure 3:**
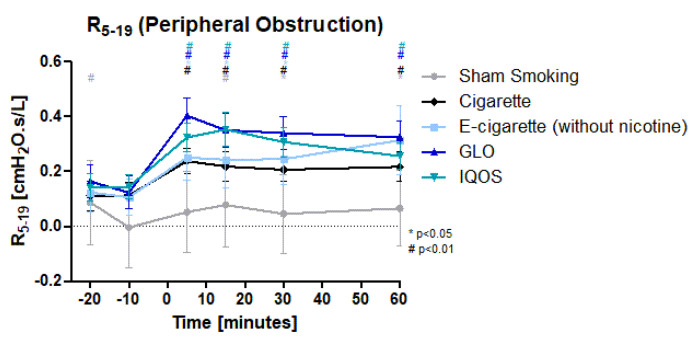
R5–R19 (Peripheral obstruction): R5–R19 as a marker of peripheral obstruction is displayed over time. Values are normally distributed. *t*-tests were performed, comparing each value to the baseline value (−10 min) of the respective group.

Peripheral obstruction (Figure 3) was highly significantly increased in Cig, IQOS, and GLO after intervention until 60 min post intervention. E-Cig (-) showed a significant increase for 30 min. The maximum increase was reached after 5 min in GLO, Cig, and E-cig (-) and after 15 min in IQOS and Sham. Again, increases in HTP products (IQOS 124.4%/144%) and GLO 226.6%) were higher than in Cig (93%) and Sham. With 136%, E-cig (-) increase was similar to IQOS. 5 min and 15 min after the intervention, IQOS and GLO differed significantly from Sham. GLO was also significantly different to Cig after 5 min. Sham smoking showed a highly significant increase at only 15 min post intervention, remaining significant until 60 min post intervention.

**Figure 4:**
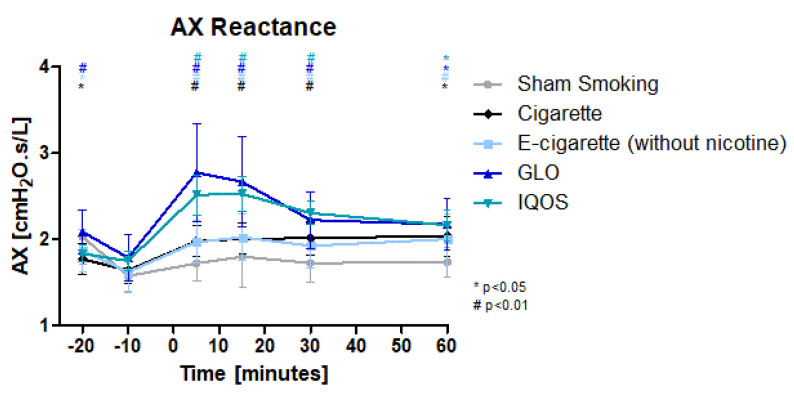
AX (Reactance area): Reactance area is displayed over time. Values are normally distributed. *t*-tests were performed, comparing each value to the baseline value (−10 min) of the respective group.

AX (reactance area) (Figure 4) was highly significantly increased in all devices, but no significant changes could be detected in Sham. The maximum increase was reached after 5 min in GLO, after 15 min in E-cig (-), IQOS, and Sham and after 60 min in Cig. The increase in Cig and E-cig (-) was similar with 20,4% (max 24.1%) (Cig) and 21.4% (max 24,8%) (E-cig (-)). In HTP products we could see higher increases of 43.8% (max 44.5%) (IQOS) and 55.2% (GLO). The difference was most prominent 5 min and 15 min post intervention. After 30 min the difference was significant between IQOS and Sham. The increase remained highly significant for 30 min in IQOS, GLO, and Cig and until the 60 min measurement in E-Cig (-).

**Figure 5:**
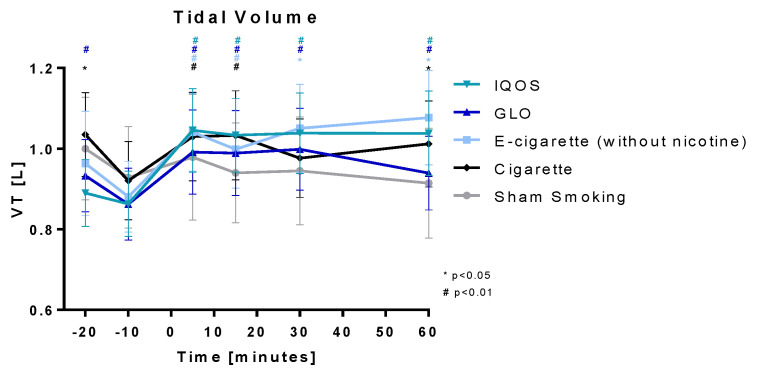
Tidal volume: Tidal volume is displayed over time. Values are normally distributed. *t*-tests were performed, comparing each value to the baseline value (−10 min) of the respective group.

Tidal volume (Figure 5) was significantly increased for 15 min after the intervention in all devices. The increase remained highly significant for 60 min in IQOS and GLO and significant in E-cig (-). The initial increase was highest in IQOS (21.1%) and E-cig (-) (18.2%/22.3% after 60 min), followed by GLO (15%/15.8% after 30 min), Cig (11.8%/12.2% after 15 min), and Sham (5.4%). No significant changes were seen in Sham.

### 3.3. Heart Rate, Peripheral, and Central Blood Pressure and Pulse Pressure

**Figure 6:**
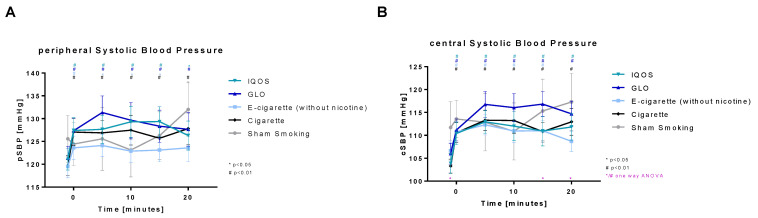
(**A**) Peripheral and (**B**) central systolic blood pressure. Peripheral and central systolic blood pressure are displayed over time. Values are normally distributed. *t*-tests were performed, comparing each value to the baseline value (−10 min) of the respective group. One-way ANOVA was performed to detect significant differences between groups at the respective time.

Both peripheral and central systolic blood pressure (Figure 6) were highly significantly increased after consumption of nicotine-containing devices (Cig, IQOS, and GLO). In peripheral systolic blood pressure, the increase stayed highly significant for 20 min in Cig and GLO and for 15 min in IQOS (significant after 20 min). An increase was also detected in E-Cig (-), which was highly significant for 5 min and significant for 15 min. In sham smoking a significant elevation occurred only 20 min after the intervention. In central systolic blood pressure, all groups but sham smoking were highly significantly increased for 15 min (E-cig (-)) and 20 min (Cig, IQOS, and GLO), respectively. Compared to peripheral, the central systolic blood pressure was lower (central: 103 to 119 mmHG, peripheral: 117 to 132 mmHG).

**Figure 7:**
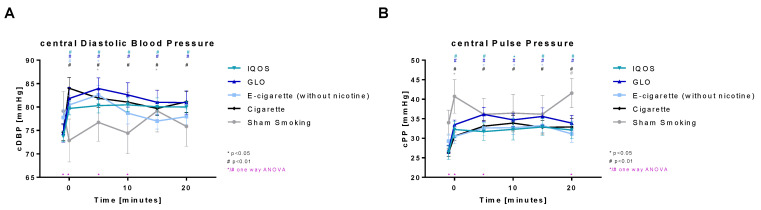
(**A**) Central diastolic blood pressure (**B**) central pulse pressure. Central diastolic blood pressure and pulse pressure are displayed over time. Values are normally distributed. *t*-tests were performed, comparing each value to the baseline value (−10 min) of the respective group. One-way ANOVA was performed to detect significant differences between groups at the respective time.

Central diastolic blood pressure (Figure 7A) was highly significantly increased in all nicotine-containing devices (Cig, IQOS, and GLO) directly after consumption. The increase stayed highly significant for more than 20 min. E-cig (-) also showed an initial highly significant increase that was significant after 5 min. No significant alterations were detected at later time points. Sham was also significantly increased after 5 and 15 min. Immediately before, until 10 min after the intervention, all groups differed significantly from each other.

Central pulse pressure (Figure 7B) was highly significantly increased directly after consumption of nicotine-containing devices (Cig, IQOS, and GLO) for more than 20 min. (After 10 min, the IQOS increase was significant). E-cig (-) was significantly increased 5 to 15 min after the intervention. Sham was significantly increased directly after the intervention and again significantly increased 20 min post intervention.

**Figure 8:**
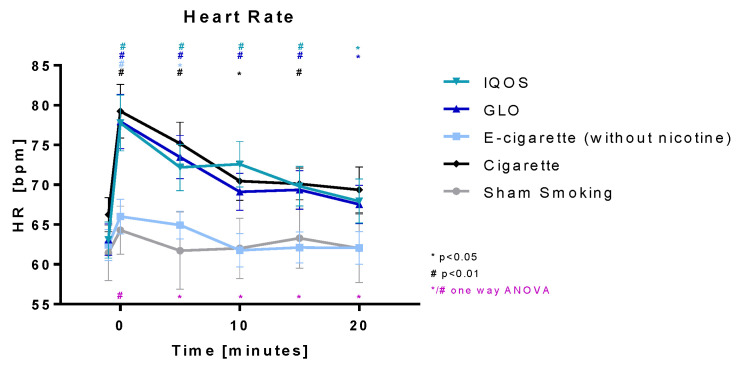
Heart rate: Heart rate is displayed over time. Values are normally distributed. *t*-tests were performed, comparing each value to the baseline value (−10 min) of the respective group. One-way ANOVA was performed to detect significant differences between groups at the respective time.

Heart rate (Figure 8) was highly significantly increased directly after the intervention in all devices. Highly significant elevation lasted for 15 min in Cig, IQOS, and GLO. In E-cig (-), heart rate was significantly increased after 5 min but returned to baseline after 10 min. An increase in IQOS (23.3%) and GLO (23.5%) was similar to Cig (19.6%). In E-cig (-), the increase was less distinct (5.8%). No significant changes could be detected in the sham smoking group. Right after the intervention all five groups differed significantly from each other and stayed significantly different until 30 min post intervention.

### 3.4. Parameters of Arterial Stiffness

**Figure 9:**
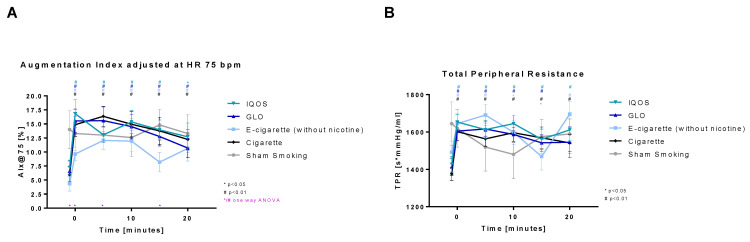
(**A**) Augmentation index adjusted at heart rate of 75 bpm and (**B**) total peripheral resistance. Augmentation index and total peripheral resistance are displayed over time. Values are normally distributed. *t*-tests were performed, comparing each value to the baseline value (−10 min) of the respective group. One-way ANOVA was performed to detect significant differences between groups at the respective time.

The augmentation index adjusted at HR 75 bpm (Figure 9A) shows a highly significant increase directly after the intervention in all nicotine-containing devices (Cig, IQOS, GLO) and is also highly significantly increased after the consumption of nicotine-free e-cigarettes. The increase stays highly significant for 20 min in Cig, GLO, and E-Cig (-) and 15 min in IQOS. In sham smoking, no significant change in the augmentation index was detected. Directly before and after the intervention, as well as 5 and 15 min post intervention, all groups differed significantly from each other.

Total peripheral resistance (Figure 9B) is highly significantly increased after consumption of nicotine-containing devices (Cig, IQOS, and GLO) for 20 min (Cig and IQOS) and 15 min (GLO), respectively. After consumption of E-cig (-) total peripheral resistance is also highly significantly increased after 5 min and significantly increased after 10 min. After 20 min another highly significant increase was detected. Sham was increased significantly only after 15 min.

## 4. Discussion

Heated tobacco products are currently heavily promoted by tobacco companies as a modern and less harmful alternative to cigarette smoking [35,36]. While shortly after the market launched, many studies about HTP were funded by the producing tobacco companies themselves [37], recently, more independent studies about heated tobacco products have been performed, which turned out to be more critical concerning their health impact [16,17,19]. We performed a crossover study, comparing acute effects on small airway function and arterial stiffness after consumption of HTP, cigarettes, nicotine-free e-cigarettes and sham smoking. Following HTP consumption we detected an increase in arterial stiffness as well as compromised small airways, which was similar to the effects measured after cigarette smoking.

Airwave oscillometry (AOS) of the lung showed impaired resistance and reactance immediately after the consumption of all devices except sham smoking. Resistance was increased at 5 Hz, a sign of central obstruction, and at 5–19 Hz, reflecting peripheral obstruction probably caused by acute bronchoconstriction [38]. The increased area of reactance reflects impaired lung elasticity and is a marker for a small airway disease [38]. Tobacco smoke is known to lead to both acute bronchoconstriction and chronic obstructive pulmonary disease (COPD), as well as emphysema and lung fibrosis [39]. Although HTP aerosols reportedly contain harmful substances in much smaller concentrations [17], in our study, the increase in resistance and, especially, in the reactance area was even more pronounced, albeit not significant, than after cigarette consumption. However, current data on this topic are heterogenous and in vivo studies in humans are still rare. Some studies reported similar effects of HTP and cigarettes on lung function in humans in vitro and in vivo [40,41]; another study showed similar damages to lung tissue in mice [22]. Other studies reported lower toxicity to bronchial epithelial cells after HTP exposition compared to cigarettes but higher than after e-cigarette exposure [42]. Nevertheless, despite lower concentrations of toxicants in HTP fumes, the majority of studies [19], including ours, found similar impairment of small airway function after heated tobacco products and cigarette consumption. These findings raise concerns about long-term lung damage of these products. In our study, not only did nicotine-containing devices show an increase in resistance and reactance but the nicotine-free e-cigarette also did. There is evidence that propylene glycol (PG) and vegetable glycerin in e-cigarettes lead to lung remodeling and impaired lung function [43,44]. Short-term effects on lung function were described after the consumption of nicotine-containing e-cigarettes [45]. However, other studies did not report any alterations [46], or only small alterations, in lung function after e-cigarette consumption [47]. A study investigating nicotine-free e-cigarettes found small but significant decreases in FEV1 and FEF25 [48]. Our study suggests the short-term effects of nicotine-free e-cigarettes on small airways, but quite possibly, this effect is smaller than after cigarette consumption. Since AOS provides more sensitive results concerning small airway function than spirometry, further studies on this matter using AOS could generate more insight on this topic.

As expected, hemodynamic parameters were increased after the consumption of the nicotine-containing devices cigarettes, IQOS^TM^ and GLO^TM^. Central and peripheral blood pressure as well as heart rate, augmentation index, and total peripheral resistance were significantly elevated for the nicotine half-life of 20 min. Since nicotine is known to increase blood pressure and heart rate [49,50], as well as arterial stiffness parameters [51,52,53], nicotine-related effects could explain the findings in this respect. In our study, hemodynamic parameters were equally increased after the consumption of HTP and cigarettes. This aligns with [54], whereas Yaman et al. and a previous study of our group with a similar crossover design found a less distinct increase in blood pressure after HTP consumption [23,55]. Hypertension is associated with an abundance of morbidities, such as myocardial infarction, stroke, heart failure, renal disease and even dementia [56]. Increased arterial stiffness is considered an independent risk factor for cardiovascular mortality [57]. The augmentation index can hereby serve as an early warning sign of increased vascular stiffness in younger people [58]. Our study showed similar behavior in blood pressure and arterial stiffness shortly after HTP and cigarette consumption, indicating a similar cardiovascular hazard potential of both products.

Interestingly, elevated hemodynamic parameters also followed the consumption of nicotine-free e-cigarettes but not sham smoking. These effects were shorter and less pronounced than after consumption of cigarettes or HTP but suggest additional nicotine-independent alterations in hemodynamics. Similar findings were also reported in a study by Antoniewicz et al. [59] but not in the study of Gonzalez et al. [60]. Additionally, we found significantly elevated diastolic blood pressure levels not only during nicotine half-life but for at least 50 min after cigarette and HTP consumption. Possible explanations could be prolonged nicotine effects or effects of other ingredients contained in both products. Since elevated diastolic blood pressure levels are considered a separate cardiovascular risk factor [61], it might be interesting to check for repeatability in future studies.

The transferability of this study is clearly limited by the rather small sample size of 17 test subjects. However, with our crossover design and randomized testing order of the devices, we could strongly reduce selection bias. Also, as a control group we used both nicotine-free e-cigarettes and sham smoking. Since we could detect the effects of nicotine-free e-cigarettes on respiratory and hemodynamic parameters, we considered the sham smoking group the more suitable control group and recommended it for future studies. Still, single outliers influenced our results, especially in the sham smoking group, which consisted of only seven participants. Finally, our study was designed to only investigate the acute effects of heated tobacco products and does not provide evidence of the long-term consequences of HTP.

When speaking of the risks of heated tobacco product use, it is also relevant to consider its range of applications. It has become consensual, that HTP consumption is harmful and addictive [12]. Knowing the individual and societal consequences of cigarette smoking, HTP consumption can clearly not be recommended to a “nicotine naive” population. Tobacco harm reduction includes alternatives to cigarette smoking that need to be satisfying enough for their users to permanently switch to a health risk as low as possible. Nicotine replacement therapy is important in assisting smoking cessation. It provides users with consistent nicotine doses to relieve cravings [5]. These products do not have any adverse health effects except for the nicotine-caused effect and provide low addictive potential but raise long-term smoking cessation rates only by 50–60% [13]. Handling and nicotine kinetics in e-cigarettes and heated tobacco products are similar to cigarettes. This could explain why these products are considered more satisfying by their users [62]. However, e-cigarettes are addictive, and consumption is considered a health risk itself [62,63,64]. The consequences of heated tobacco products seem to be similar and have been discussed in detail above. Still, more evidence concerning short- and long-term health consequences is required. A recommendation for heated tobacco products as a substitute product for cigarette smoking should only follow a critical benefit-risk consideration. However, our study suggests that the risks may outweigh the benefits.

## 5. Conclusions

Satisfying substitute devices could be a useful tool in smoking cessation. However, it is important that these devices cause as little harm as possible. Seemingly containing a lot fewer harmful substances than cigarettes, heated tobacco products (HTP) have been promoted as a promising device in this field. Investigating their acute impact on small airway function and arterial stiffness, in our study, we could not find any evidence for a harm reduction in HTP compared to cigarettes and nicotine-free e-cigarettes. Hemodynamic parameters were elevated in a nicotine-related manner that was equivalent to cigarettes, leading to the conclusion that HTP cause short-term elevated arterial stiffness and acute cardiovascular stress, associating these products with increased cardiovascular risk. Furthermore, when investigating small airway function, we even detected an increase in resistance and reactance in IQOS^TM^ and GLO^TM^ exceeding the acute effects of cigarettes and nicotine-free e-cigarettes, which, although not significant, could be an interesting subject for further investigations. To conclude, our findings support that consumption of heated tobacco products is associated with acute worsening of arterial stiffness and increased stiffness of small airways. Whether these findings also apply to regular smokers needs to be determined, as detected changes might be different in a possibly pre-damaged respiratory and cardiovascular system. Still, to further understand the health impact of these products, more independent studies that investigate short- and long-term effects are needed.

## Figures and Tables

**Table 1:** Specifies baseline characteristics for all subjects.

Sex	All (*n* = 17)	Male (*n* = 9)	Female (*n* = 8)	*p*-Value
Age [years]	24.2 ± 1.1	25.2 ± 4.6	23 ± 4.4	0.329
Weight [kg]	71.3 ± 3.8	82.9 ± 11.5	58.3 ± 5.6	<0.01
Height [cm]	177 ± 2.7	184.8 ± 9.0	168.4 ± 5.7	<0.01
BMI [kg/m^2^]	22.5 ± 0.6	24.2 ± 1.9	20.5 ± 1.6	0.01
Waist [cm]	77.5 ± 2.7	86.1 ± 5.5	67.9 ± 7.0	<0.01
Hip [cm]	91.2 ± 2.1	96.7 ± 5.5	85.1 ± 7.1	0.02
Cigarettes per day	1.12 ± 0.8	1.2 ± 0.7	1.0 ± 1.0	0.687
Fagerström Test for Nicotine Dependence [points]	0.0 ± 0.0	0.0 ± 0.0	0.0 ± 0.0	

## Data Availability

Not applicable.

## References

[1] Bundesministerium für Gesundheit Rauchen. https://www.bundesgesundheitsministerium.de/service/begriffe-von-a-z/r/rauchen.html.

[2] Institute of General Practice, Addiction Research and Clinical Epidemiology Unit DEBRA Study. Prävalenz Aktueller Tabak-Raucher*innen in Deutschland. https://www.debra-study.info/.

[3] Pashutina Y., Kastaun S., Kotz D. *DEBRA Factsheet 5*; Die Motivation zum Rauchstopp Skala. Düsseldorf, Germany. https://www.google.com.tw/url?sa=t&rct=j&q=&esrc=s&source=web&cd=&cad=rja&uact=8&ved=2ahUKEwiL16mRu5WBAxU5o1YBHRuGB9UQFnoECBYQAQ&url=https%3A%2F%2Fwww.debra-study.info%2Fwp-content%2Fuploads%2F2022%2F02%2FFactsheet-05-v7-1.pdf&usg=AOvVaw0PqllTq-Dz1zFksTZjBw2M&opi=89978449.

[4] Hughes J.R., Keely J., Naud S. (2004). Shape of the relapse curve and long-term abstinence among untreated smokers. Addiction.

[5] Stead L.F., Perera R., Bullen C., Mant D., Hartmann-Boyce J., Cahill K., Lancaster T. (2018). Nicotine replacement therapy for smoking cessation. Cochrane Database Syst. Rev..

[6] Hartmann-Boyce J., McRobbie H., Lindson N., Bullen C., Bergh R., Theodoulou A., Notley C., Rigotti N.A., Turner T., Butler A.R. (2022). Electronic cigarettes for smoking cessation. Cochrane Database Syst. Rev..

[7] Kotz D., Jackson S., Brown J., Kastaun S. (2022). The Effectiveness of E-Cigarettes for Smoking Cessation. Dtsch. Arztebl. Int..

[8] Bhatt J.M., Ramphul M., Bush A. (2020). An update on controversies in e-cigarettes. Paediatr. Respir. Rev..

[9] Gotts J.E., Jordt S.-E., McConnell R., Tarran R. (2019). What are the respiratory effects of e-cigarettes?. BMJ.

[10] Chun L.F., Moazed F., Calfee C.S., Matthay M., Gotts J. (2017). Pulmonary toxicity of e-cigarettes. Am. J. Physiol. Lung Cell Mol. Physiol..

[11] Lüdicke F., Picavet P., Baker G., Haziza C., Poux V. (2018). Effects of Switching to the Menthol Tobacco Heating System 2.2, Smoking Abstinence, or Continued Cigarette Smoking on Clinically Relevant Risk Markers: A Randomized, Controlled, Open-Label, Multicenter Study in Sequential Confinement and Ambulatory Settings (Part 2). Nicotine Tob. Res..

[12] Goodall S., Gale N., Thorne D., Haziza C., Hadley S., Prasad K., Gilmour I., Miazzi F., Proctor C. (2022). Evaluation of behavioural, chemical, toxicological and clinical studies of a tobacco heated product glo™ and the potential for bridging from a foundational dataset to new product iterations. Toxicol. Rep..

[13] WHO Heated Tobacco Products Information Sheet. https://www.who.int/publications/i/item/WHO-HEP-HPR-2020.2.

[14] Centers for Disease Control and Prevention (US), National Center for Chronic Disease Prevention and Health Promotion, Office on Smoking and Health (US) (2010). How Tobacco Smoke Causes Disease: The Biology and Behavioral Basis for Smoking-Attributable Disease: A Report of the Surgeon General.

[15] Başaran R., Güven N.M., Eke B.C. (2019). An Overview of iQOS^®^ as a New Heat-Not-Burn Tobacco Product and Its Potential Effects on Human Health and the Environment. Turk. J. Pharm. Sci..

[16] Schaller K., Kahnert D.-B.S., Mons U. (2020). E-Zigaretten und Tabakerhitzer—Ein Überblick.

[17] Dusautoir R., Zarcone G., Verriele M., Garcon G., Fronval I., Beauval N., Allorge D., Riffault V., Locoge N., Lo-Guidic J.-M. (2021). Comparison of the chemical composition of aerosols from heated tobacco products, electronic cigarettes and tobacco cigarettes and their toxic impacts on the human bronchial epithelial BEAS-2B cells. J. Hazard. Mater..

[18] Tattan-Birch H., Jackson S.E., Dockrell M., Brown J. (2022). Tobacco-free Nicotine Pouch Use in Great Britain: A Representative Population Survey 2020–2021. Nicotine Tob. Res..

[19] Znyk M., Jurewicz J., Kaleta D. (2021). Exposure to Heated Tobacco Products and Adverse Health Effects, a Systematic Review. Int. J. Environ. Res. Public Health.

[20] Vivarelli F., Canistro D., Cirillo S., Elias R., Granata S., Mussoni M., Burattini S., Falcieri E., Turrini E., Fimognari C. (2021). Unburned Tobacco Cigarette Smoke Alters Rat Ultrastructural Lung Airways and DNA. Nicotine Tob. Res..

[21] Nitta N.A., Sato T., Komura M., Yoshikawa H., Suzuki Y., Mitsui A., Kuwasaki E., Takahashi F., Kodama Y., Seyama K. (2022). Exposure to the heated tobacco product IQOS generates apoptosis-mediated pulmonary emphysema in murine lungs. Am. J. Physiol. Lung Cell Mol. Physiol..

[22] Bhat T.A., Kalathil S.G., Leigh N., Muthumalage T., Rahman I., Goniewicz M., Thanvala Y. (2021). Acute Effects of Heated Tobacco Product (IQOS) Aerosol Inhalation on Lung Tissue Damage and Inflammatory Changes in the Lungs. Nicotine Tob. Res..

[23] Franzen K.F., Belkin S., Goldmann T., Reppel M., Watz H., Mortensen K., Drömann D. (2020). The impact of heated tobacco products on arterial stiffness. Vasc. Med..

[24] Benthien J., Meusel M., Cayo Talavera S., Eitel I., Drömann D., Franzen K. (2022). JUUL™ ing and Heating Lead to a Worsening of Arterial Stiffness. Medicines.

[25] van Bortel L.M., Duprez D., Starmans-Kool M.J., E Safar M., Giannattasio C., Cockcroft J., Kaiser D., Thuillez C. (2002). Clinical applications of arterial stiffness, Task Force III: Recommendations for user procedures. Am. J. Hypertens..

[26] Philip Morris Products S.A. User Guide IQOS 2.4 PLUS. www.iqos.com/gb/en/get-support/iqos-2-4-plus-guide.html.

[27] British American Tobacco S.A. Instruction Manual GLO. https://www.discoverglo.com/de/de/blog/tabak-erhitzen-anfaengerfehler/.

[28] Farsalinos K.E., Romagna G., Tsiapras D., Kyrzopoulos S., Voudris V. (2013). Evaluation of electronic cigarette use (vaping) topography and estimation of liquid consumption: Implications for research protocol standards definition and for public health authorities’ regulation. Int. J. Environ. Res. Public Health.

[29] Thoracic Medical Systems Inc. (2018). Tremoflo C-100 Airwave Oscillometry System User Manual English.

[30] Porojan-Suppini N., Fira-Mladinescu O., Marc M., Tudorache E., Oancea C. (2020). Lung Function Assessment by Impulse Oscillometry in Adults. Ther. Clin. Risk Manag..

[31] Hallett S., Toro F., Ashurst J.V. (2023). Physiology, Tidal Volume.

[32] Weber T., Wassertheurer S., Rammer M., Maurer E., Hametner B., Mayer C., Kropf J., Eber B. (2011). Validation of a brachial cuff-based method for estimating central systolic blood pressure. Hypertension.

[33] Wassertheurer S., Kropf J., Weber T., van der Giet M., Baulmann J., Ammer M., Hametner B., Mayer C., Eber B., Magometschnigg D. (2010). A new oscillometric method for pulse wave analysis: Comparison with a common tonometric method. J. Hum. Hypertens..

[34] Hukkanen J., Jacob P., Benowitz N.L. (2005). Metabolism and disposition kinetics of nicotine. Pharmacol. Rev..

[35] Philip Morris Products S.A. Philip Morris International, Website. Unsere Visionen, eine Rauchfreie Zukunft Gestalten. https://www.pmi.com/markets/germany/de/%C3%BCber-uns/our-vision.

[36] British American Tobacco (Germany) GmbH glo Website. https://www.discoverglo.com/de/de/.

[37] Simonavicius E., McNeill A., Shahab L., Brose L. (2019). Heat-not-burn tobacco products: A systematic literature review. Tob. Control.

[38] Desiraju K., Agrawal A. (2016). Impulse oscillometry: The state-of-art for lung function testing. Lung India.

[39] Christenson S.A., Smith B.M., Bafadhel M., Putcha N. (2022). Chronic obstructive pulmonary disease. Lancet.

[40] Sohal S.S., Eapen M.S., Naidu V.G.M., Sharma P. (2019). IQOS exposure impairs human airway cell homeostasis: Direct comparison with traditional cigarette and e-cigarette. ERJ Open Res..

[41] Pataka A., Kotoulas S., Chatzopoulos E., Grigoriou I., Sapalidis K., Kosmidis C., Vagionas A., Perdikouri E.-I. (2020). Acute Effects of a Heat-Not-Burn Tobacco Product on Pulmonary Function. Medicina.

[42] Leigh N.J., Tran P.L., O’Connor R.J., Goniewicz M. (2018). Cytotoxic effects of heated tobacco products (HTP) on human bronchial epithelial cells. Tob. Control.

[43] Madison M.C., Landers C.T., Gu B.-H., Chang C.-Y., Tung H.-Y., You R., Hong M., Baghaei N., Song L.-Z., Porter P. (2019). Electronic cigarettes disrupt lung lipid homeostasis and innate immunity independent of nicotine. J. Clin. Investig..

[44] Szafran B.N., Pinkston R., Perveen Z., Ross M., Morgan T., Paulsen D.B., Penn A., Kaplan B., Noel A. (2020). Electronic-Cigarette Vehicles and Flavoring Affect Lung Function and Immune Responses in a Murine Model. Int. J. Mol. Sci..

[45] Vardavas C.I., Anagnostopoulos N., Kougias M., Evangelopoulou V., Connoly G., Behrakis P. (2012). Short-term pulmonary effects of using an electronic cigarette: Impact on respiratory flow resistance, impedance, and exhaled nitric oxide. Chest.

[46] Flouris A.D., Chorti M.S., Poulianiti K.P., Jamurtas A.Z., Kostikas K., Tzatzarakis M., Hayes A., Tsatsaki A. (2013). Acute impact of active and passive electronic cigarette smoking on serum cotinine and lung function. Inhal. Toxicol..

[47] Unverdorben M., Mostert A., Munjal S., van der Bijl A., Potgieter L., Venter C., Liang Q., Meyer B., Roethig H.-J. (2010). Acute effects of cigarette smoking on pulmonary function. Regul. Toxicol. Pharmacol..

[48] Ferrari M., Zanasi A., Nardi E., Labate A., Ceriana P., Balestrino A., Pisani L., Corcione N., Nava S. (2015). Short-term effects of a nicotine-free e-cigarette compared to a traditional cigarette in smokers and non-smokers. BMC Pulm. Med..

[49] Benowitz N.L. (1997). The role of nicotine in smoking-related cardiovascular disease. Prev. Med..

[50] Omvik P. (1996). How smoking affects blood pressure. Blood Press..

[51] Adamopoulos D., Argacha J.-F., Gujic M., Preumont N., Degaute J.-P., van de Borne P. (2009). Acute effects of nicotine on arterial stiffness and wave reflection in healthy young non-smokers. Clin. Exp. Pharmacol. Physiol..

[52] Mahmud A., Feely J. (2004). Effects of passive smoking on blood pressure and aortic pressure waveform in healthy young adults--influence of gender. Br. J. Clin. Pharmacol..

[53] Jatoi N.A., Jerrard-Dunne P., Feely J., Mahmud A. (2007). Impact of smoking and smoking cessation on arterial stiffness and aortic wave reflection in hypertension. Hypertension.

[54] Majek P., Jankowski M., Brożek G.M. (2023). Acute health effects of heated tobacco products: Comparative analysis with traditional cigarettes and electronic cigarettes in young adults. ERJ Open Res..

[55] Yaman B., Akpınar O., Kemal H.S., Cerit L., Yüksek Ü., Söylemez N., Duygu H. (2021). Comparison of IQOS (heated tobacco) and cigarette smoking on cardiac functions by two-dimensional speckle tracking echocardiography. Toxicol. Appl. Pharmacol..

[56] Williams B., Mancia G., Spiering W., Rosei E., Azizi M., Burnier M., Clement D., Coca A., Simone G., Dominiczak A. (2018). 2018 ESC/ESH Guidelines for the management of arterial hypertension. Eur. Heart J..

[57] Laurent S., Boutouyrie P., Asmar R., Gautier I., Laloux B., Guize L., Ducimetiere P., Benetos A. (2001). Aortic stiffness is an independent predictor of all-cause and cardiovascular mortality in hypertensive patients. Hypertension.

[58] Baulmann J., Nürnberger J., Slany J., Schmieder R., Schmidt-Trucksäss A., Baumgart D., Cremerius P., Hess O., Mortensen K., Weber T. (2010). Arterielle Gefässsteifigkeit und Pulswellenanalyse. Dtsch. Med. Wochenschr..

[59] Antoniewicz L., Brynedal A., Hedman L., Lundbäck M., Bosson J. (2019). Acute Effects of Electronic Cigarette Inhalation on the Vasculature and the Conducting Airways. Cardiovasc. Toxicol..

[60] Gonzalez J.E., Cooke W.H. (2021). Acute effects of electronic cigarettes on arterial pressure and peripheral sympathetic activity in young nonsmokers. Am. J. Physiol. Heart Circ. Physiol..

[61] Flint A.C., Conell C., Ren X., Banki N.M., Chan S.L., Rao V., Melles R., Bhatt D. (2019). Effect of Systolic and Diastolic Blood Pressure on Cardiovascular Outcomes. N. Engl. J. Med..

[62] Hajek P., Pittaccio K., Pesola F., Smith K., Phillips-Waller A., Przulj D. (2020). Nicotine delivery and users’ reactions to Juul compared with cigarettes and other e-cigarette products. Addiction.

[63] Staudt M.R., Salit J., Kaner R.J., Hollmann C., Crystal R. (2018). Altered lung biology of healthy never smokers following acute inhalation of E-cigarettes. Respir. Res..

[64] Rubinstein M.L., Delucchi K., Benowitz N.L., Ramo D. (2018). Adolescent Exposure to Toxic Volatile Organic Chemicals From E-Cigarettes. Pediatrics.

